# Supplementary figures and images for: Ataxin-2, Twenty-four, and Dicer-2 are components of a noncanonical cytoplasmic polyadenylation complex
Source: Life Sci Alliance. 2022 Sep 16;5(12):e202201417. doi: 10.26508/lsa.202201417 (PMC9481931; doi:10.26508/lsa.202201417)

Figure 1A

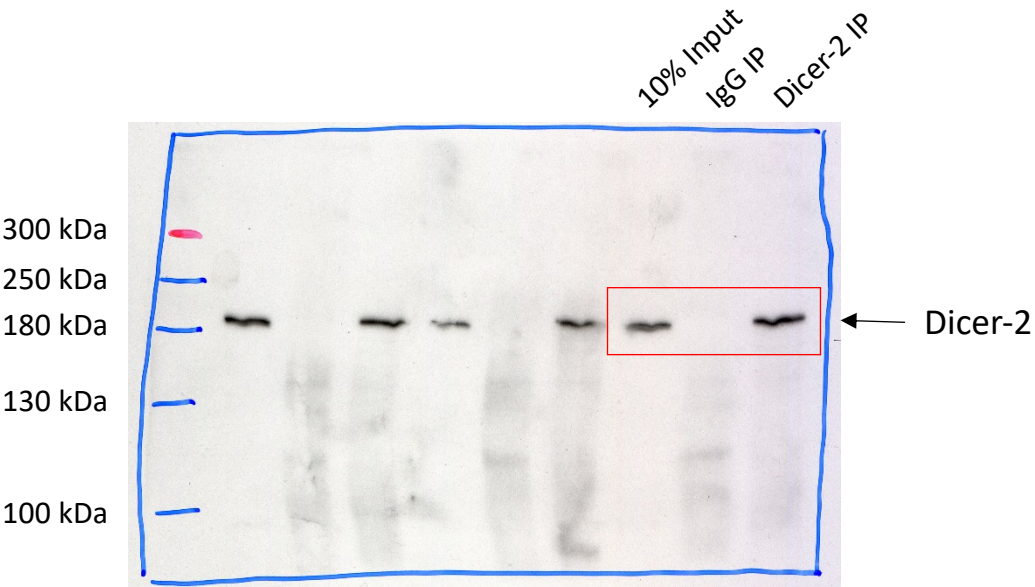

Supplement: Supplementary file 1 [file LSA-2022-01417_SdataF1.1.pdf]

Figure 4A (same MW ladder used in all runs)

Grapes:

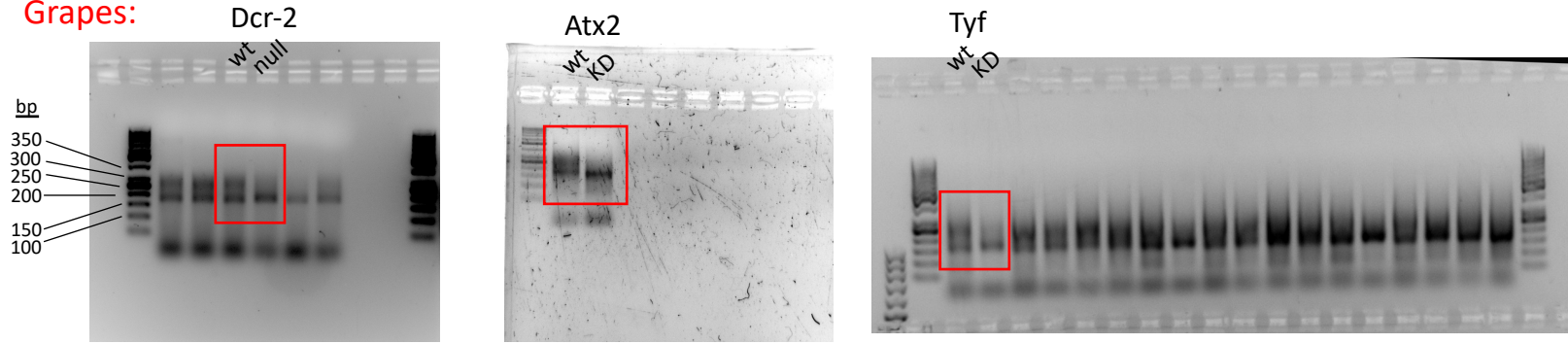

String:

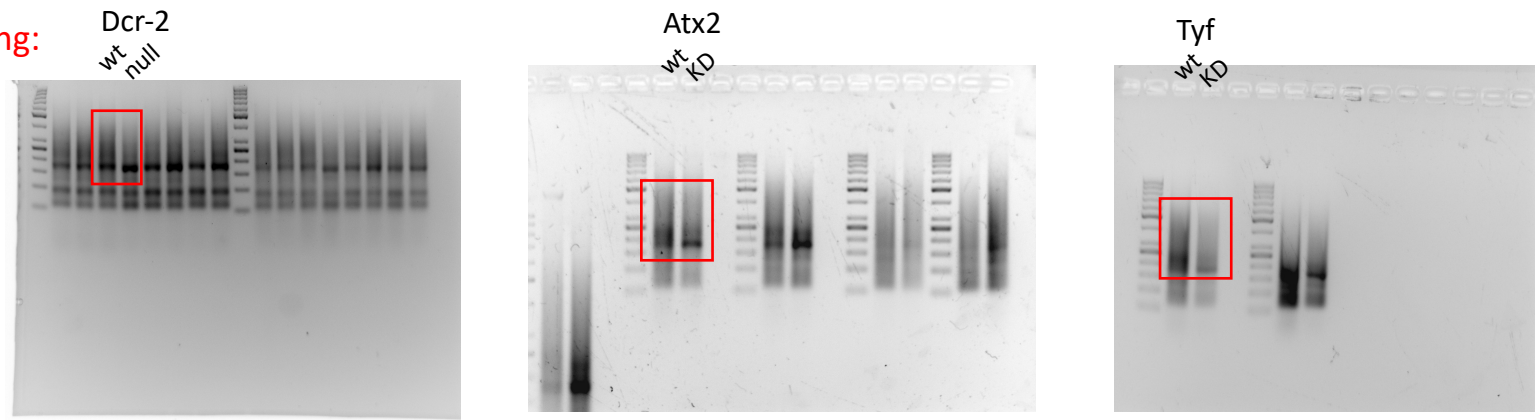

CG8180:

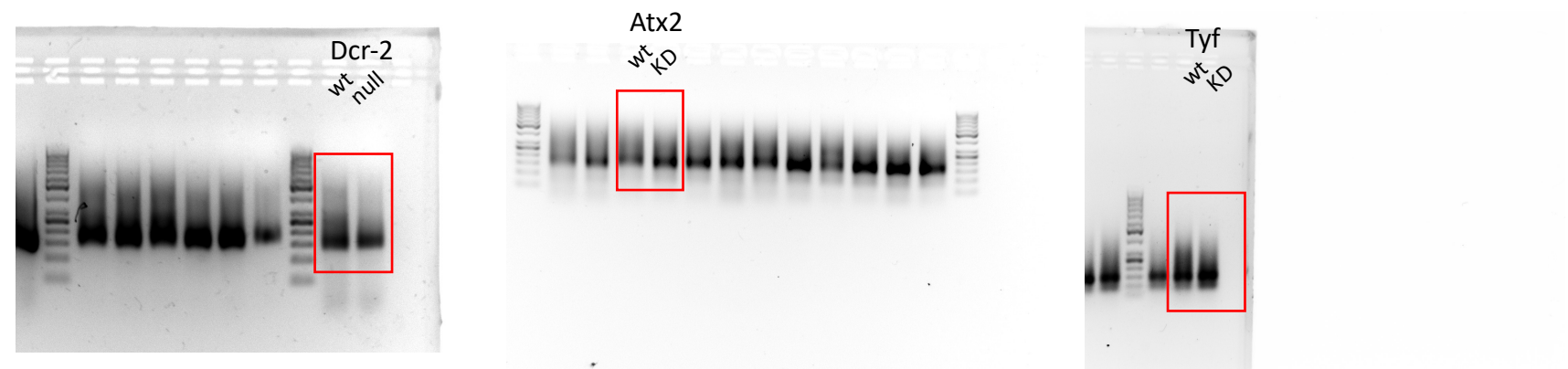

Figure 4A

EDTP:

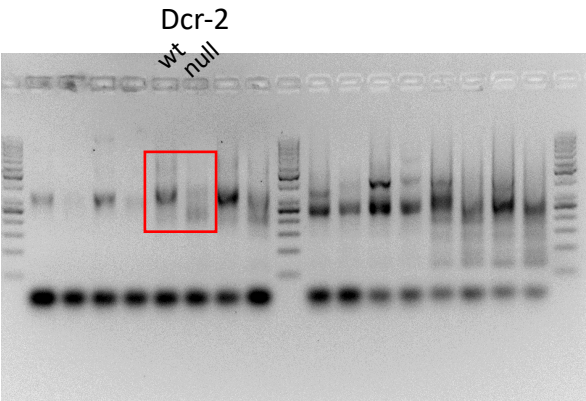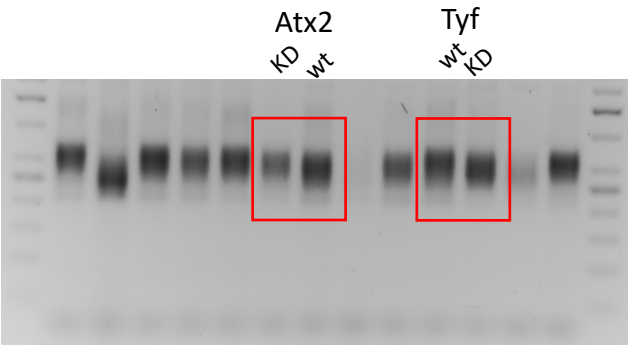

lok:

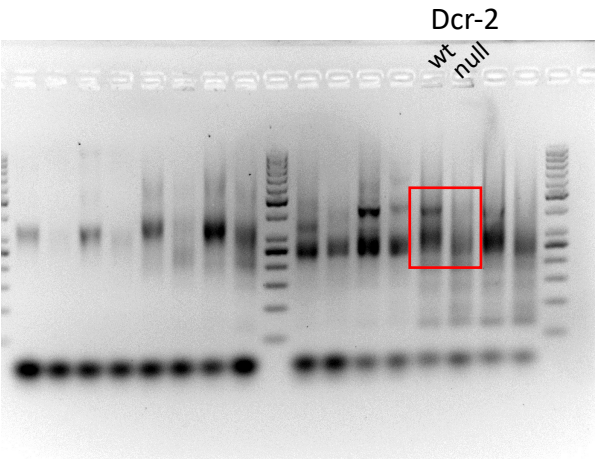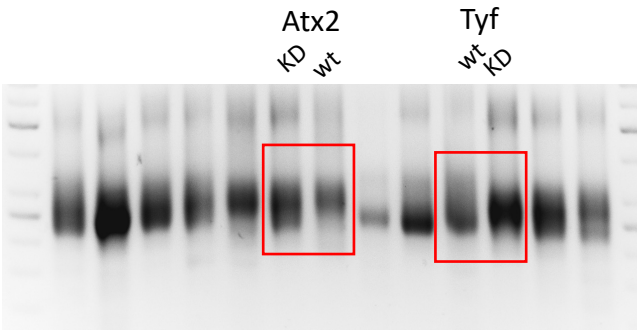

Supplement: Supplementary file 7 [file LSA-2022-01417_SdataF4.1.pdf]
