# Supplementary material for: Ataxin-2, Twenty-four, and Dicer-2 are components of a noncanonical cytoplasmic polyadenylation complex
Source: Life Sci Alliance. 2022 Sep 16;5(12):e202201417. doi: 10.26508/lsa.202201417 (PMC9481931; doi:10.26508/lsa.202201417)
Supplement: Supplementary file 4 [file LSA-2022-01417_SdataF2.pdf]

Figure 2B

1st. Immunoblot α-Wispy

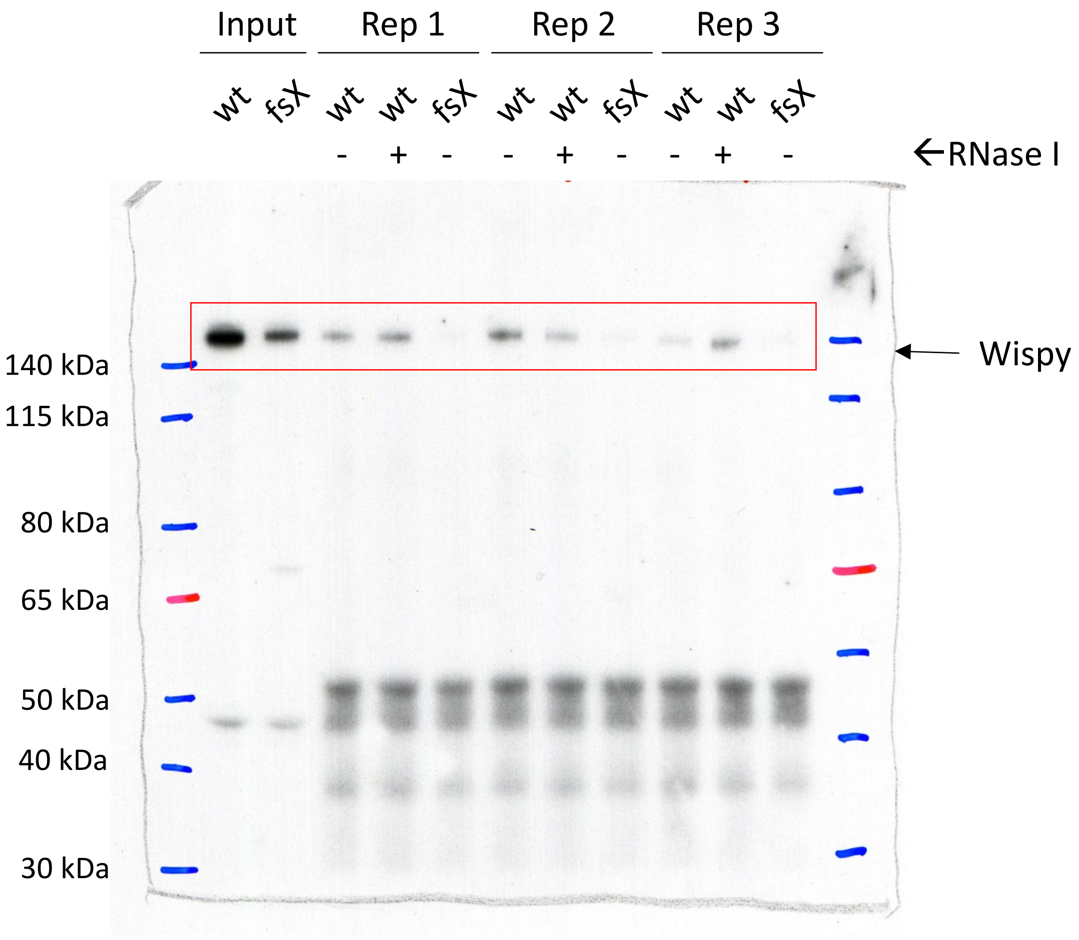

2nd. Immunoblot α-Dicer-2 (membrane was cut)

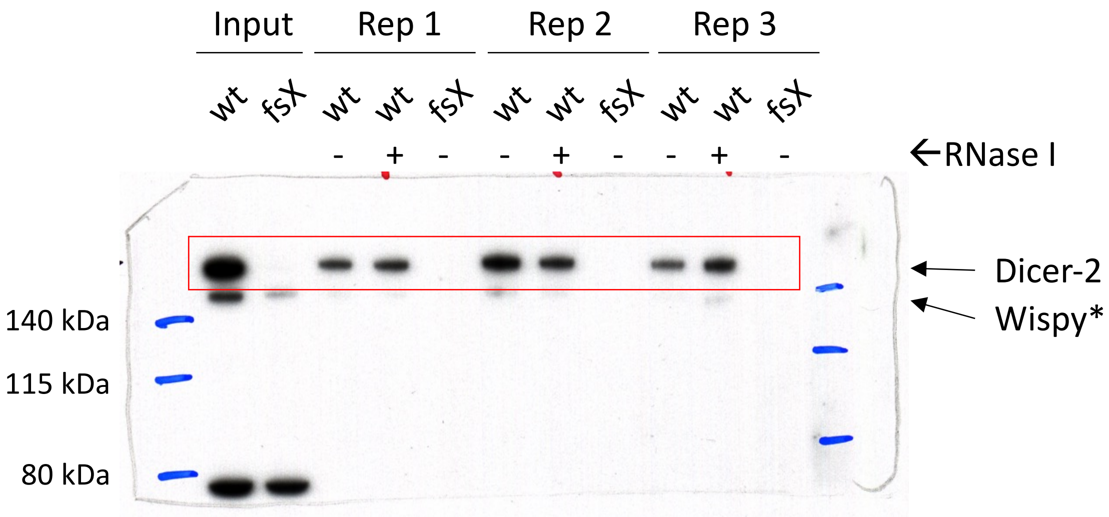

*Residual signal from the previous immunoblot against Wispy

Figure 2C

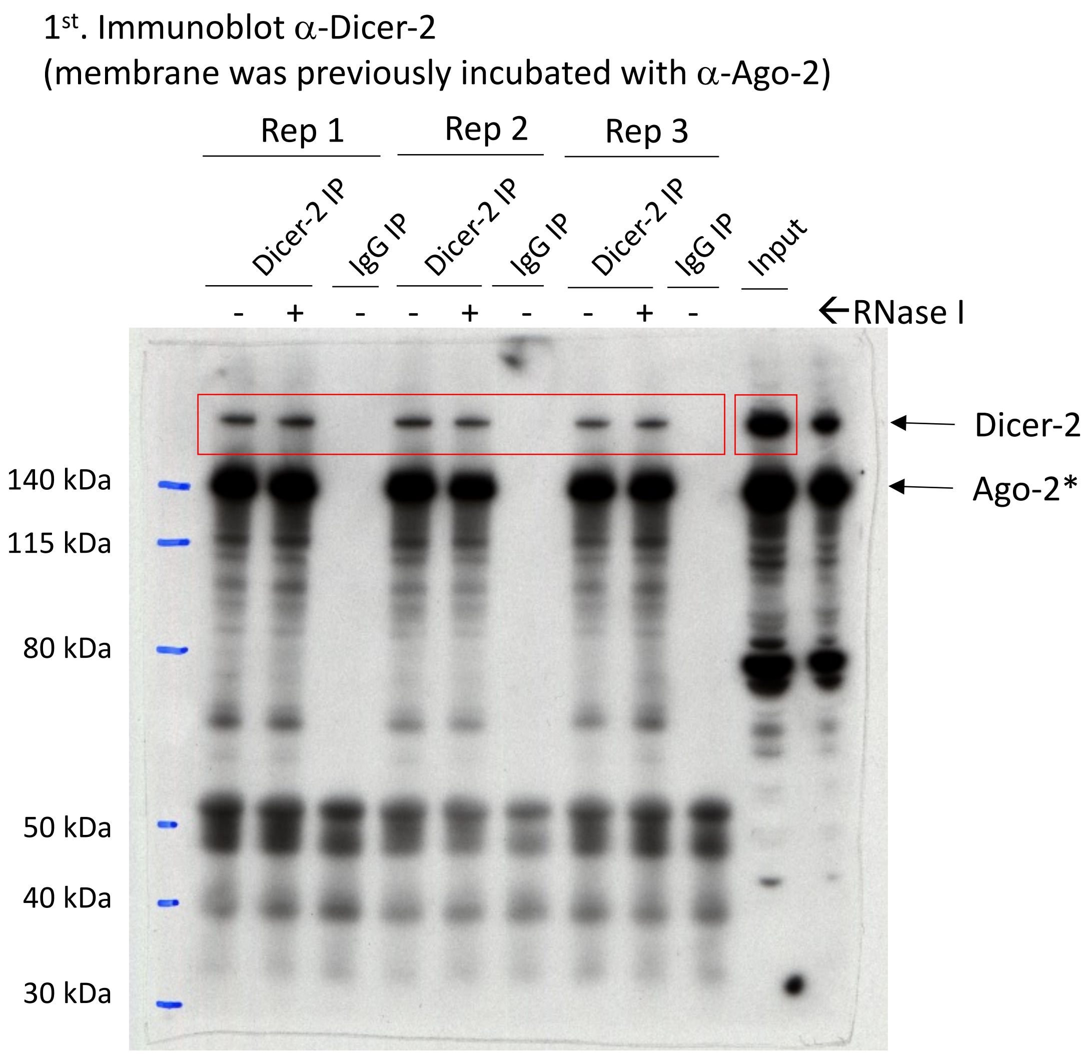

*Residual signal from a previous immunoblot against Ago-2

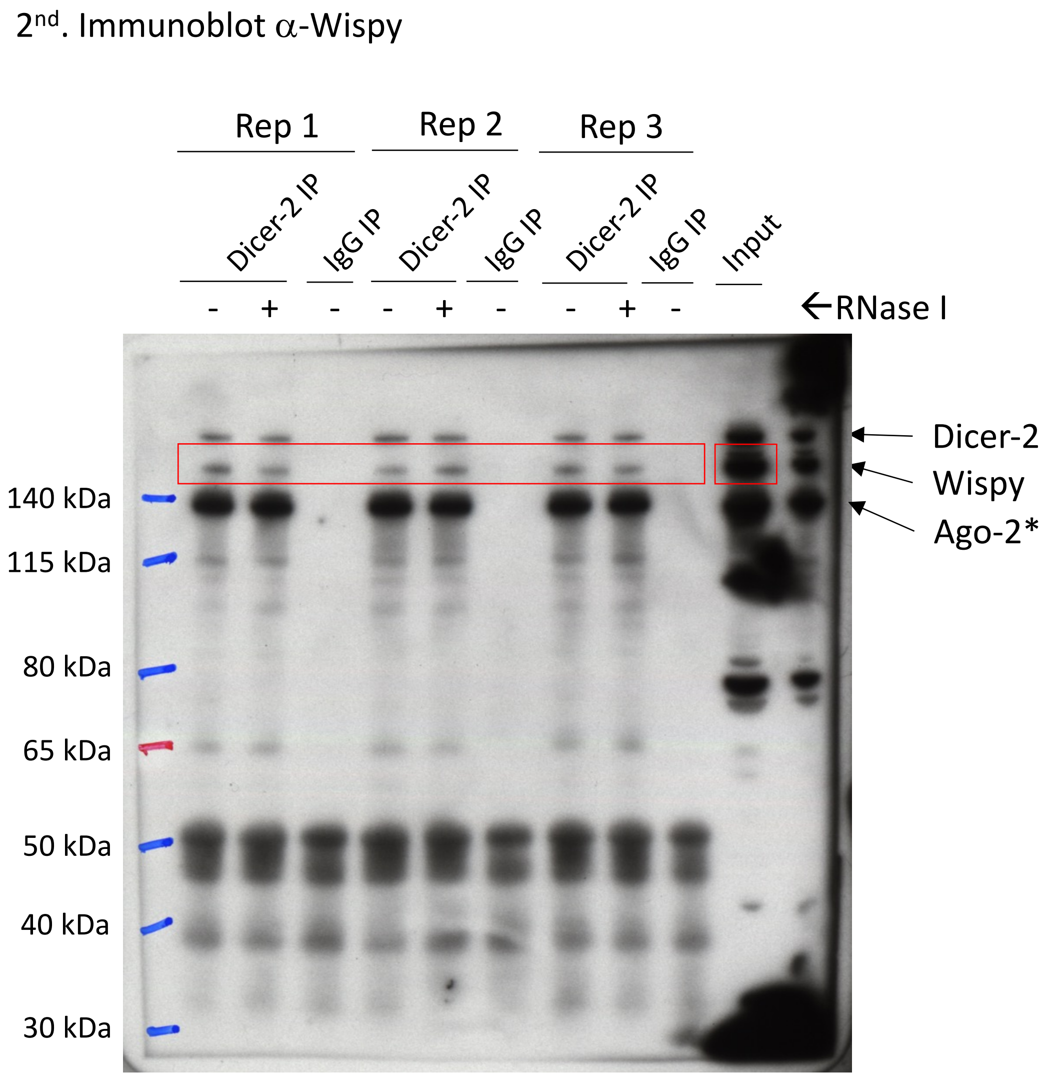

*Residual signal from a previous immunoblot against Ago-2
