# Supplementary material for: Ataxin-2, Twenty-four, and Dicer-2 are components of a noncanonical cytoplasmic polyadenylation complex
Source: Life Sci Alliance. 2022 Sep 16;5(12):e202201417. doi: 10.26508/lsa.202201417 (PMC9481931; doi:10.26508/lsa.202201417)
Supplement: Supplementary file 6 [file LSA-2022-01417_SdataF3.pdf]

Figure 3B

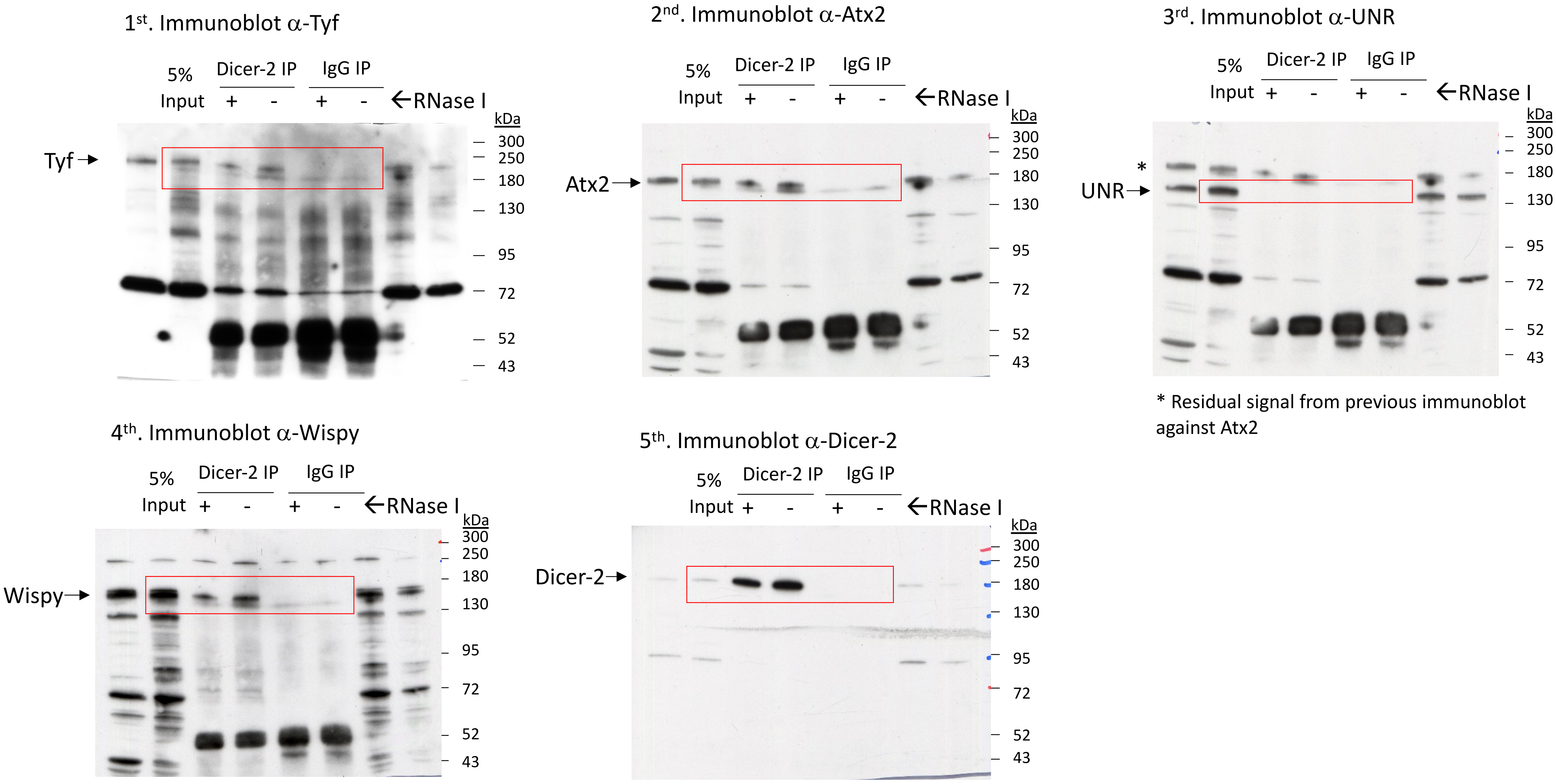
1st. Immunoblot α-Tyf
5% Input
Dicer-2 IP
IgG IP
+ - + -
←RNase I
kDa
300
250
180
130
95
72
52
43
Tyf→
2nd. Immunoblot α-Atx2
5% Input
Dicer-2 IP
IgG IP
+ - + -
←RNase I
kDa
300
250
180
130
95
72
52
43
Atx2→
3rd. Immunoblot α-UNR
5% Input
Dicer-2 IP
IgG IP
+ - + -
←RNase I
kDa
300
250
180
130
95
72
52
43
UNR→
*
* Residual signal from previous immunoblot against Atx2
4th. Immunoblot α-Wispy
5% Input
Dicer-2 IP
IgG IP
+ - + -
←RNase I
kDa
300
250
180
130
95
72
52
43
Wispy→
5th. Immunoblot α-Dicer-2
5% Input
Dicer-2 IP
IgG IP
+ - + -
←RNase I
kDa
300
250
180
130
95
72
52
43
Dicer-2→

Figure 3C

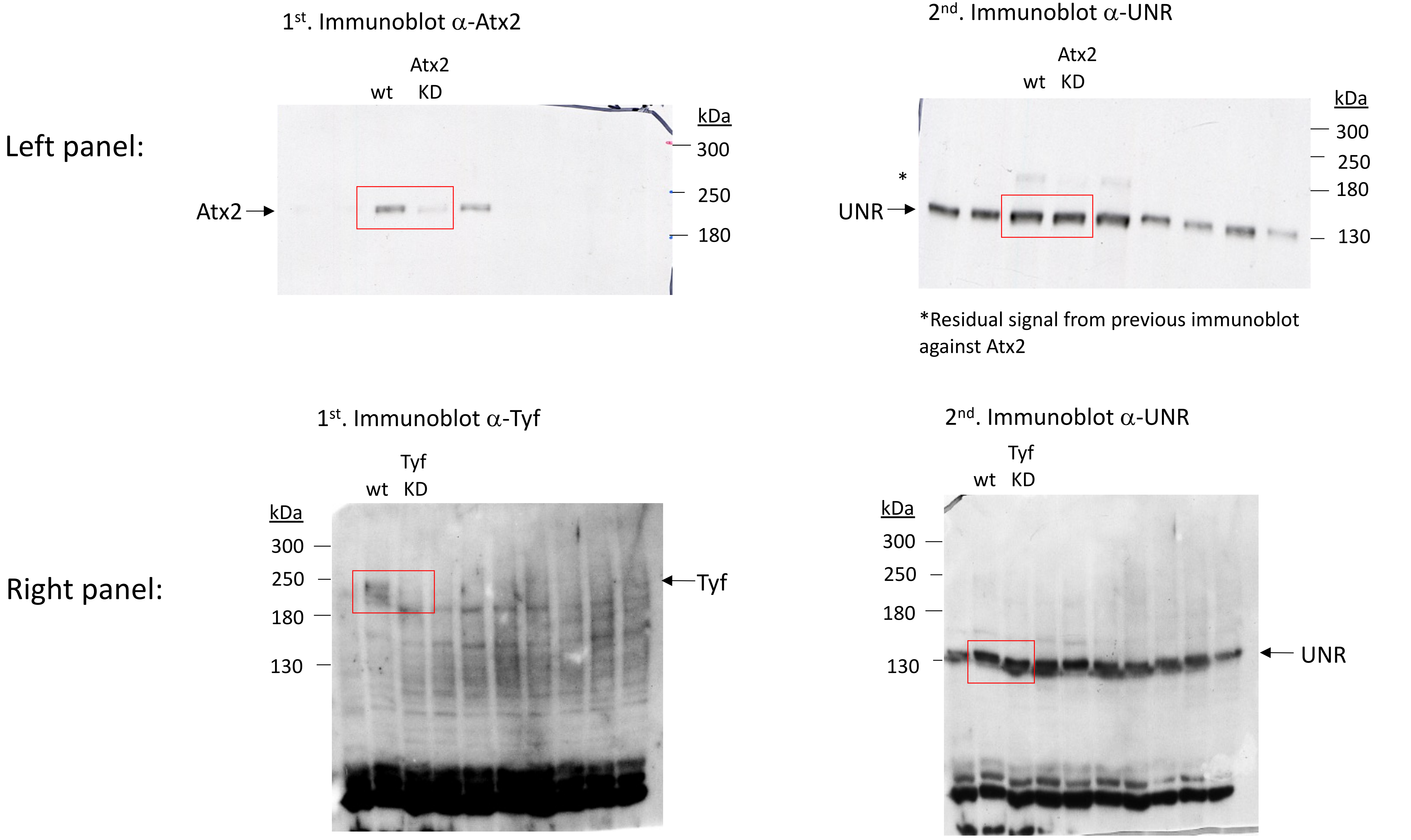
Left panel:
1st. Immunoblot α-Atx2
Atx2
wt KD
kDa
300
250
180
Atx2
2nd. Immunoblot α-UNR
Atx2
wt KD
kDa
300
250
180
130
*
UNR
*Residual signal from previous immunoblot against Atx2
Right panel:
1st. Immunoblot α-Tyf
Tyf
wt KD
kDa
300
250
180
130
Tyf
2nd. Immunoblot α-UNR
Tyf
wt KD
kDa
300
250
180
130
UNR

Figure 3D

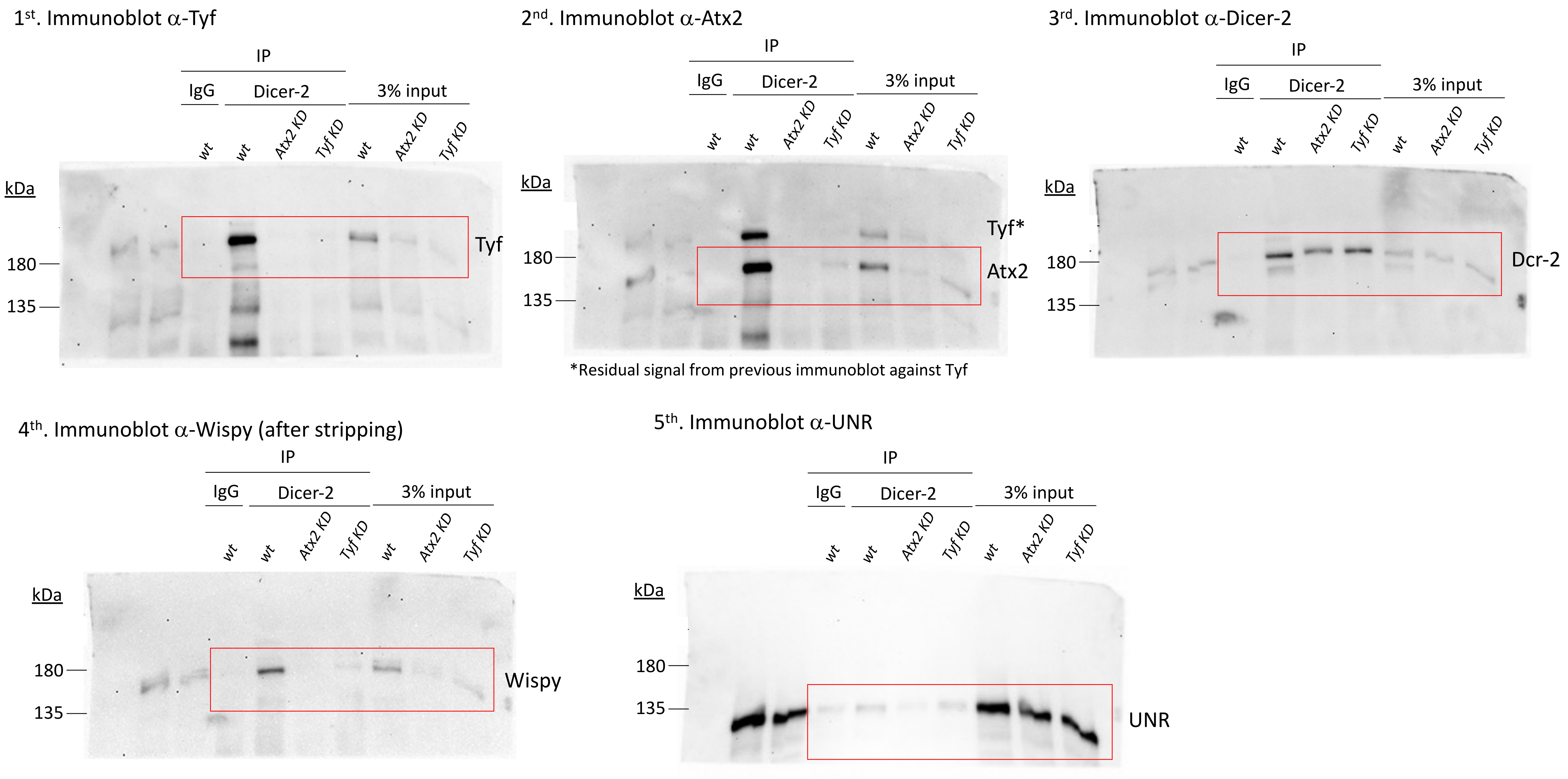

1st. Immunoblot α-Tyf
IP
IgG
Dicer-2
3% input
wt
wt
Atx2 KD
Tyf KD
wt
Atx2 KD
Tyf KD
kDa
180
135
Tyf
2nd. Immunoblot α-Atx2
IP
IgG
Dicer-2
3% input
wt
wt
Atx2 KD
Tyf KD
wt
Atx2 KD
Tyf KD
kDa
180
135
Tyf*
Atx2
*Residual signal from previous immunoblot against Tyf
3rd. Immunoblot α-Dicer-2
IP
IgG
Dicer-2
3% input
wt
wt
Atx2 KD
Tyf KD
wt
Atx2 KD
Tyf KD
kDa
180
135
Dcr-2
4th. Immunoblot α-Wispy (after stripping)
IP
IgG
Dicer-2
3% input
wt
wt
Atx2 KD
Tyf KD
wt
Atx2 KD
Tyf KD
kDa
180
135
Wispy
5th. Immunoblot α-UNR
IP
IgG
Dicer-2
3% input
wt
wt
Atx2 KD
Tyf KD
wt
Atx2 KD
Tyf KD
kDa
180
135
UNR

Figure 3E

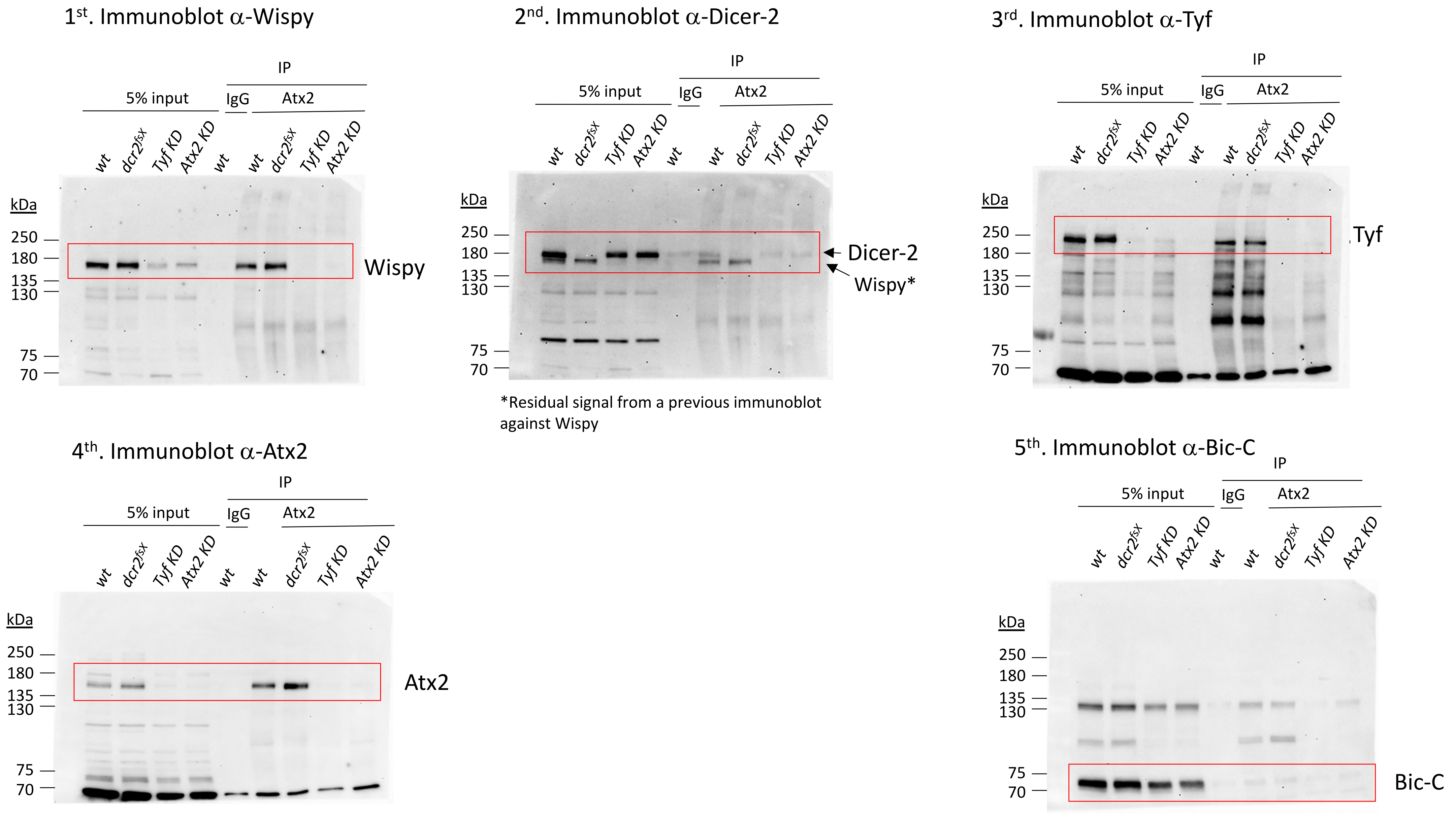

*Residual signal from a previous immunoblot against Wispy
